# Effects of various debonding and adhesive clearance methods on enamel surface: an in vitro study

**DOI:** 10.1186/s12903-017-0349-6

**Published:** 2017-02-27

**Authors:** Xiao-Chuan Fan, Li Chen, Xiao-Feng Huang

**Affiliations:** 1 Department of Stomatology, Beijing Friendship Hospital, Capital Medical University, No.95 Yong’an Road, Xicheng District, Beijing, 100050 China; 2 Department of Orthodontics, Capital Medical University School of Stomatology, No.4 Tiantan Xili, Dongcheng District, Beijing, 100050 China

**Keywords:** Orthodontic debonding, Adhesive clearance, Enamel damage, Resin modified glass ionomer cement (RMGIC), Surface roughness, Scanning electron microscope (SEM)

## Abstract

**Background:**

The purpose of this study was to evaluate orthodontic debonding methods by comparing the surface roughness and enamel morphology of teeth after applying two different debonding methods and three different polishing techniques.

**Methods:**

Forty eight human maxillary premolars, extracted for orthodontic reasons, were randomly divided into three groups. Brackets were bonded to teeth with RMGIC (Fuji Ortho LC, GC, Tokyo, Japan) (two groups, *n* = 18 each) after acid etching (30s), light cured for 40 s, exposed to thermocycling, then underwent 2 different bracket debonding methods: debonding pliers (Shinye, Hangzhou, China) or enamel chisel (Jinzhong, Shanghai, China); the third group (*n* = 12) comprised of untreated controls, with normal enamel surface roughness. In each debonded group, three cleanup techniques (*n* = 6 each) were tested, including (I) diamond bur (TC11EF, MANI, Tochigi, Japan) and One-Gloss (Midi, Shofu, Kyoto, Japan), (II) a Super-Snap disk (Shofu, Kyoto, Japan), and (III) One-Gloss polisher. The debonding methods were compared using the modified adhesive remnant index (ARI, 1–5). Cleanup efficiencies were assessed by recording operating times. Enamel surfaces were qualitatively and quantitatively evaluated with scanning electron microscopy (SEM) and surface roughness tester, respectively. Two surface roughness variables were evaluated: Ra (average roughness) and Rz (10-point height of irregularities).

**Results:**

The ARI scores of debonded teeth were similar with debonding pliers and enamel chisel (Chi-square = 2.19, *P* > 0.05). There were significant differences between mean operating time in each group (*F* = 52.615, *P* < 0.01). The diamond bur + One-Gloss took the shortest operating time (37.92 ± 3.82 s), followed by the Super-Snap disk (56.67 ± 7.52 s), and the One-Gloss polisher (63.50 ± 6.99 s). SEM appearance provided by the One-Gloss polisher was the closest to the intact enamel surface, and surface roughness (Ra: 0.082 ± 0.046 μm; Rz: 0.499 ± 0.200 μm) was closest to the original enamel (Ra: 0.073 ± 0.048 μm; Rz: 0.438 ± 0.213 μm); the next best was the Super-Snap disk (Ra: 0.141 ± 0.073 μm; Rz: 1.156 ± 0.755 μm); then, the diamond bur + One-Gloss (Ra: 0.443 ± 0.172 μm; Rz: 2.202 ± 0.791 μm).

**Conclusions:**

Debonding pliers were safer than enamel chisels for removing brackets. Cleanup with One-Gloss polisher provided enamel surfaces closest to the intact enamel, but took more time, and Super-Snap disks provided acceptable enamel surfaces and efficiencies. The diamond bur was not suitable for removing adhesive remnant.

## Background

The acid etch technique and direct bonding technique, first described by Buonocore [1] in 1955 and Newman [2] in 1965, were major improvements in daily orthodontic practice. Once orthodontic therapies with fixed appliances are completed, the bonded brackets and residual adhesive must be removed. Ideally, the debonding procedure would lead to restitution of the tooth enamel, or at least, the enamel surface could be restored, as closely as possible, to its original state [3, 4]. However, when the bracket debonding process is inadequate, the enamel is injured, which results cracks and fractures in the enamel surface. This condition leads tooth sensitivity, and it increases the risk of caries and pulp inflammation [5, 6]. Therefore, it is important to evaluate an appropriate debonding process for removing brackets used in orthodontic practice. Restoration of the enamel after orthodontic treatment includes two major steps: debonding and enamel surface polishing.

Previous studies reported different methods for removing brackets, such as mechanical methods; chemical solvents; ultrasonic scalars; and lasers [7–12]. Among these methods, the mechanical removal methods are most widely used in clinical practice. Successful debonding process relies on maintaining an intact enamel structure without producing iatrogenic damage. Bishara suggested that excessive debonding strength (>11.3 MPa) may cause enamel cracks, and that cracks are less likely to appear with lower forces (7.3 MPa) [13]. Moreover, Su and colleagues established three standardized bracket removal techniques, which are commonly applied in the clinic [8]. Those results suggested that lifting forces are more acceptable for clinical use than shearing forces. Brosh and colleagues compared in vivo the bracket debonding forces of two different debonding techniques [7]. In one technique, the blades of pliers were placed between the wings and base of the bracket (wings model); in the other technique, the pliers’ blades were placed between the base of the bracket and the enamel surface (base model). The results indicated that lower loads were required with the wings model and tensile forces developed at the interface with this technique. Zarrinnia et al. also demonstrated that bracket-removing pliers can apply a pulling force on the bracket wing, which avoids unnecessary torque on the tooth, when the beak rested on the labial enamel surface [14]. However, those studies lacked an evaluation of the micro-structure of the enamel surface after removing the adhesive.

In previous studies, numerous finishing and polishing procedures were tested, including a diamond bur, a tungsten carbide bur, polishing cups with pumice, and ultrasonic scalars [14–18]. Most recently, new enamel cleanup methods, such as sand blasting, polishing disks, silicone dioxide particle polishers, and a Nd: YAG laser, have been introduced into the orthodontics clinic [4, 14, 18–23]. The diamond finishing bur or tungsten carbide bur was a highly efficient (least time-consuming) surface cleaning method. However, burs can produce grooves and superimposed abrasion marks on the enamel [4, 14, 24, 25]. Therefore, these kinds of instruments require following secondary polishing treatment [26, 27]. One-Gloss polishers, could provide good tooth polishing after bracket debonding [21, 22]. However, Ye et al. reported that more color change occurred when One-Gloss polishers were used for polishing after carbide bur debonding [28]. Bonetti et al. [20] and Zarrinia et al. [14] suggested that removing resin with a 12-bladed tungsten carbide bur, followed by polishing with Sof-Lex disks, could produce the smoothest enamel surface. With scanning electron microscope (SEM) images, the smoothest surface was obtained when Sof-Lex disks were used alone [4]. Tufekci reported that the enamel loss was not significantly different between the Sof-Lex disk and tungsten carbide bur methods [29].

To our knowledge, most previous studies only focused on one stage of removing fixed appliances after orthodontic treatment (debonding or enamel surface polishing), and mostly performed subjective evaluations of SEM images without a quantitative evaluation that is necessary for accurate comparisons. Few studies described the removal of resin modified glass ionomer cement (RMGIC), although is commonly used in clinic. Due to the bonding properties of RMGIC, two curing systems may be applied. One is the polymerization of composite resin, which is similar to the method used for resin adhesives. The second is an acid–base reaction [30, 31], which is different from that used for resin adhesives.

Therefore, our objective is to compare the surface roughness and enamel morphology of teeth after applying two different debonding methods and three different polishing techniques, and to identify the most appropriate, efficient method of debonding after orthodontic treatments with fixed appliances.

## Methods

### Specimen preparation

A total of 48 human maxillary premolars were extracted from 12- to 18-year-old patients for orthodontic reasons. The study has been approved by the ethics committee of Beijing Stomatological Hospital. Guardians of all patients provided written authorizations for use of the extracted teeth in this study. To determine eligibility for the study, teeth were examined under 10× magnification with a stereomicroscope (SMZ-1500, NIKON, Tokyo, Japan). This study included only teeth that had intact buccal enamel, no surface cracks from extraction forces, no caries, and no exposure to chemical agents (e.g., H_2_O_2_). All selected teeth were cleaned with flowing water and tissue debris were removed with scalpel. Then, teeth were stored in a 0.5% (weight/volume) chloramine-T solution at room temperature to inhibit bacterial growth for 24 h. After that, all specimens were stored in distilled water at 4 °C, which was refreshed weekly, for a maximum of 6 months after extraction.

### Bonding and thermocycling

The buccal surfaces of each crown were cleaned with fluoride-free pumice, then sprayed with water, and dried for 10s. Out of 48 specimens, 12 served as untreated controls, and 36 were treated with an etching procedure with 35% phosphoric acid gel (Gluma, Heraeukulzer, Hanau, Germany) for 30s. After etching, teeth were rinsed with water for 30s and dried with an oil-free stream until the etched surfaces appeared chalky white. Before bonding, the enamel surfaces of etched teeth were moistened with distilled water. RMGIC bonding materials (Fuji Ortho LC, GC, Tokyo, Japan) were handled according to the manufacturers’ instructions. Stainless steel maxillary premolar brackets (0.024-in. twin, TOMY, Tokyo, Japan) were placed at the Facial Axis point (FA) on the midbuccal surface of the tooth along the Facial Axis of the clinical crown (FACC). The RMGIC adhesive were light-cured for a total of 40 s, which included 10s each at the mesial, distal, gingival, and occlusal margins. The light source came from a curing light CL-628 (Beyond, Houston, USA). The original bulb was used with wavelength of 400–480 nm and a minimum output light intensity of 900 mW/cm^2^. All bonding procedures were carried out by a single operator, with a standard technique.

After storing the specimens in water at 37 ± 2 °C for 24 h, thermocycling was performed for 500 cycles from 5 to 55 °C, with a dwell time of 20s; the transfer time between the two baths was 5–10s. This procedure followed the recommendations of the International Organization for Standardization [32].

### Debonding process and evaluation of ARI

The specimens bonded with brackets (*n* = 36) were randomly divided into two groups of 18 specimens. One group was debonded with orthodontic debonding pliers (Shinye, Hangzhou, China). The blades of the pliers were placed between the wings and the base of the bracket, according to the ‘wings model’ introduced by Brosh [7]. The other group was debonded with an enamel chisel (Jinzhong, Shanghai, China). The blade of the chisel was also placed between the wings and the base of the bracket. During the debonding process, an impact force transferred to the bracket through the chisel, and the instantaneous shear force causes the bracket to fall off.

After debonding, the enamel surfaces were examined at 10× magnification with a stereomicroscope. A modified adhesive remnant index (ARI), described by Bishara, was used to quantify the amount of adhesive on the tooth surface [33]. The ARI scale was: 1 = no adhesive remaining; 2 = less than 10% of adhesive remaining; 3 = 10 to 90% of adhesive remaining; 4 = over 90% of adhesive remaining; and 5 = all adhesive remaining on the tooth.

### Cleanup process

Each debonding test group was further randomly divided into three subgroups, each with 6 specimens, which received one of three cleanup methods: The first cleanup process implemented a high-speed diamond finishing bur (TC-11EF, MANI, Tochigi, Japan) cooled with water, for removing gross remnants of adhesive (only a few adhesive left on enamel surface); then, a One-Gloss polisher (Midi, Shofu, Kyoto, Japan) was used for further removal of the remaining adhesive and to polish the enamel until no visible residue left (the diamond bur + One-Gloss method). The second cleanup process implemented Super-Snap disks (Shofu, Kyoto, Japan) with three grades: the medium grade (purple disk), which was used for removing gross remnants of adhesive, and the fine (green disk) and superfine (red disk) grades, which were used to remove the remaining adhesive and to polish the enamel until no visible residue left (Super-Snap method). The third cleanup process implemented the One-Gloss polisher for removing adhesive and polishing the enamel till no visible residue left alone (One-Gloss method). The operating times required for the cleanup processes were recorded.

### Surface roughness measurements

The enamel surfaces of the three cleanup process groups and one control group (*n* = 12 in each group) were evaluated with a surface roughness taster (JB-4C, Temin Optical Instrument Corp., Shanghai, China). The roughness taster sensor was placed on the enamel surface to measure the surface roughness. Five recordings were performed over a distance of 0.25 mm that the probe travels across the enamel surface (surface distance) for each specimen. The mean values of two surface roughness variables were evaluated. The first variable was the average roughness (Ra), defined as the arithmetic mean of the absolute surface roughness, measured from the centre line, over the surface distance, after removing the shape error and any large waviness content. The Ra reflected the overall roughness of the enamel surface. The second variable was the 10-point height of irregularities (Rz), defined as the average distance between the 5 highest peaks and the 5 deepest valleys, measured from a line parallel to the mean line, over the surface distance. The Rz is a useful value, when only a short surface distance is available for assessment, such as the surface distance evaluated in this study [34].

### Scanning electron microscopy observations

Three specimens of each group were vacuum-dried in a vacuum dryer (JFD-310, JEOL, Tokyo, Japan) for 48 h at room temperature. Then, each specimen was coated with approximately 460 Å of a gold palladium mixture. The specimens were analyzed qualitatively with a SEM (S-4800, HITACHI, Tokyo, Japan), operated at 2.0KV, to evaluate the damaged area on the enamel surface.

### Statistical analysis

Statistical analysis was performed with the IBM SPSS Statistics software (Version 20.0, IBM, New York, USA). The modified ARIs of the two debonding groups were evaluated with the chi-square test to determine statistical differences. The one-way analysis of variance (ANOVA) and the Bonferroni multiple comparisons test were used to analyze statistical differences in operating times among the three cleanup groups and in surface roughness parameters (Ra, Rz) among the three cleanup groups and the control group. The significance threshold for all statistical tests was predetermined at *P* < 0.05.

**Fig. 1:**
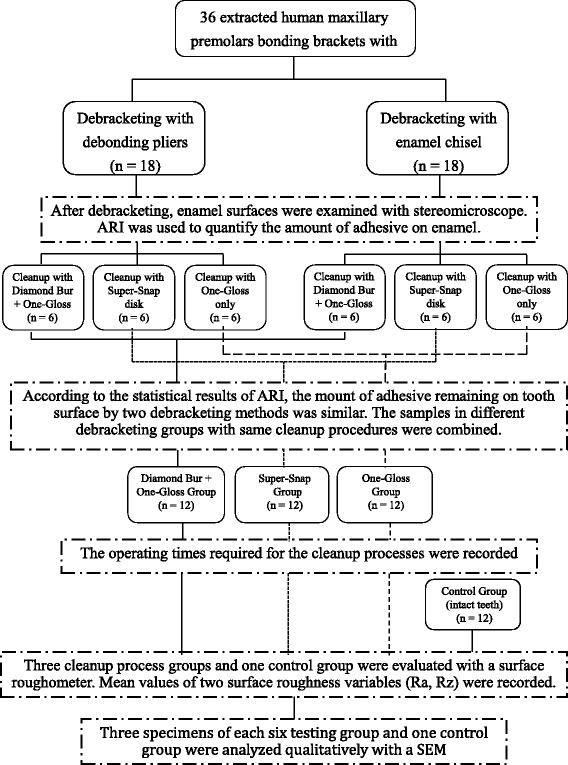
The consort type diagram of the study process and variables measured

The consort type diagram of the study process and variables measured was shown in Fig. 1.

## Results

### Adhesive remnant index

The modified ARIs remnant of the two groups treated with different debonding processes are presented in Table 1. The chi-square test showed no statistical difference between the ARIs of the two groups (*P* > 0.05).

**Table 1:** Distribution of ARI scores for different debonding methods

Group	Sample Size	ARI					Chi-square value	*P*-value
		1	2	3	4	5		
Enamel chisel	18	0	0	4	3	11	2.190	0.534
Debonding pliers	18	0	2	3	3	10		

ARI score: 1 = no adhesive remaining; 2 = less than 10% of adhesive remaining; 3 = 10 to 90% of adhesive remaining; 4 = over 90% of adhesive remaining; and 5 = all adhesive remaining on the tooth

### Operating time

Table 2 shows the time required for the cleanup process with each of the three methods tested. A one-way ANOVA of the time required for cleanup showed an *F* value of 52.615, indicating a significant difference (*P* < 0.01) between groups. A Bonferroni multiple comparisons test (Table 3) showed that the time for cleanup with the diamond bur + One-Gloss method was significantly (*P* < 0.01) shorter than that of the other two methods. Similarly, the cleanup time with the Super-Snap method was significantly (*P* < 0.05) shorter than that with the One-Gloss method.

**Table 2:** Operating times for cleanup (s)

Group	Sample Size	Mean (SD)	*F*-value	*P*-value
Diamond bur + One-Gloss	12	37.92 (3.82)	52.615	<0.001
Super-Snap	12	56.67 (7.52)		
One-Gloss	12	63.50 (6.99)

**Table 3:** Bonferroni test results for comparing operating times

Group	Group Compared	Mean difference	*P*-value	95% Confidence Interval
Diamond bur + One-Gloss	Super-Snap	−18.750	<0.001	−25.264/−12.236
	One-Gloss	−25.583	<0.001	−32.097/−19.070
Super-Snap	One-Gloss	−6.833	0.037	−13.347/−0.320

### Surface roughness measurement

The data of Ra and Rz for each cleanup process are presented in Table 4. A one-way ANOVA of the surface roughness, measured after the different cleanup processes, showed *F* values of 37.245 for the Ra and 25.158 for the Rz, indicating a significant difference among methods (*P* < 0.01). A Bonferroni multiple comparisons test (Table 5) showed that the Ra and Rz values for the diamond bur + One-Gloss method were significantly higher (*P* < 0.01) than those found with the other methods. Similarly, the Rz value for the Super-Snap method was significantly higher (*P* < 0.05) than that found for untreated controls and for teeth treated with the One-Gloss method. There were no significant differences in surface roughness among the other methods tested.

**Table 4:** Surface roughness for each cleanup process (μm)

Group	Sample Size	Mean (SD)		*F*-value (Ra/Rz)	*P*-value (Ra/Rz)
		Ra	Rz		
Control	12	0.073 (0.048)	0.438 (0.213)	37.245/25.158	<0.001/<0.001
Diamond bur + One-Gloss	12	0.443 (0.172)	2.202 (0.791)		
Super-Snap	12	0.141 (0.073)	1.156 (0.755)		
One-Gloss	12	0.082 (0.046)	0.499 (0.200)		

*Ra* average roughness, *Rz* 10-point height of irregularities

**Table 5:** Bonferroni test results for comparing Ra & Rz values

Group	Group Compared	Ra			Rz		
		Mean difference	*P*-value	95% Confidence Interval	Mean difference	*P*-value	95% Confidence Interval
Control	Diamond bur + One-Gloss	−0.370	<0.001	−0.482/−0.258	−1.765	<0.001	−2.403/−1.126
	Super-Snap	−0.068	0.609	−0.180/0.044	−0.719	0.020	−1.357/−0.080
	One-Gloss	−0.009	1.000	−0.121/0.103	−0.062	1.000	−0.700/0.577
Diamond bur + One-Gloss	Super-Snap	0.302	<0.001	0.190/0.414	1.046	<0.001	0.408/1.684
	One-Gloss	0.361	<0.001	0.249/0.473	1.703	<0.001	1.064/2.341
Super-Snap	One-Gloss	0.059	0.914	−0.053/0.171	0.657	0.041	0.018/1.295

### Scanning electron microscopy observations

**Fig. 2:**
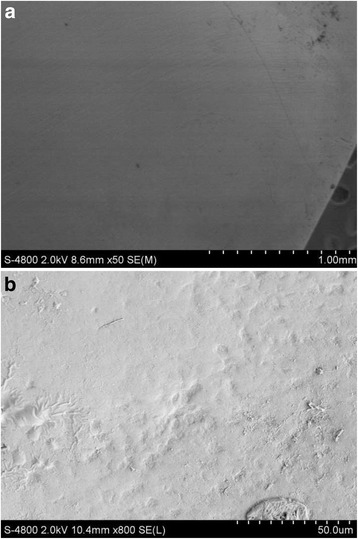
Representative scanning electron microscope (SEM) images of intact enamel surfaces. **a** SEM 50×; **b** SEM 800×

**Fig. 3:**
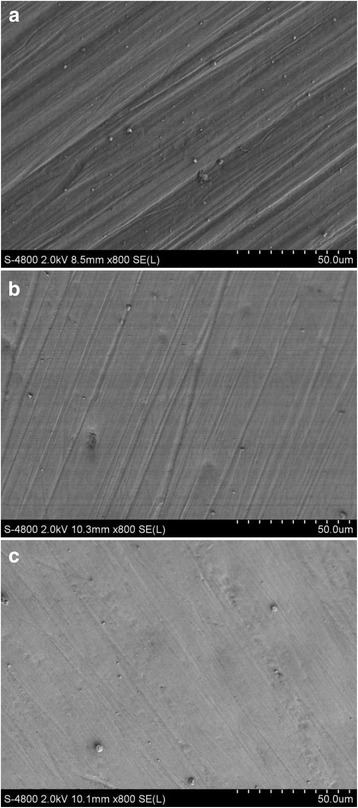
Representative scanning electron microscope (SEM) images of enamel surfaces after debonding with an enamel chisel. SEM magnification: 800×. **a** Diamond bur + One-Gloss; **b** Super-Snap; **c** One-Gloss method

**Fig. 4:**
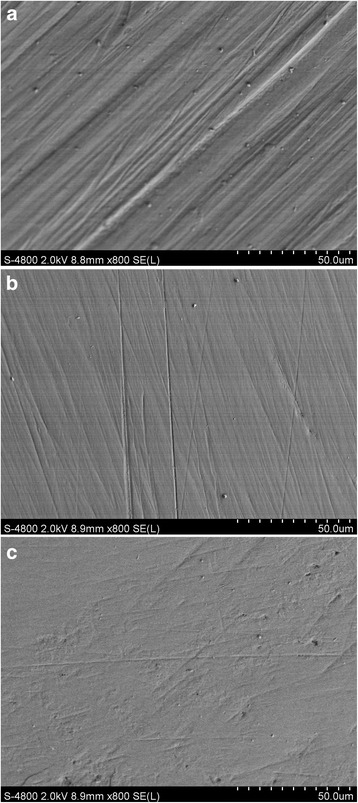
Representative scanning electron microscope (SEM) images of enamel surfaces after debonding with debonding pliers. SEM magnification: 800×. **a** Diamond bur + One-Gloss, **b** Super-Snap, **c** One-Gloss method

Figure 2 shows SEM images of the original, intact enamel. The enamel had cross-striations (Fig. 2a) and craters (Fig. 2b). Figures 3 and 4 show the enamel condition after debonding with an enamel chisel and debonding pliers, respectively, then applying the three different cleanup methods. After cleanup with the diamond bur + One-Gloss (Figs. 3a and 4a), the enamel surfaces showed the worst scratches, with thick, deep grooves and scars. After cleanup with the Super-Snap method (Figs. 3b and 4b), the enamel surfaces were smoother, with shallow scratches, and deep scars were rare. After cleanup with the One-Gloss method (Figs. 3c and 4c) the enamel surface was smoothest, with only a few, shallow scratches.

## Discussion

### Debonding process

The principle of mechanical removal method is to make a stress fracture at the enamel-adhesive-bracket interface. In the present study, we chose debonding pliers and enamel chisel for removing brackets by using pulling and shear forces respectively, and explored the effects of these two types of forces on the enamel surfaces. The modified ARI with five gradesis an internationally recognized scale for evaluating resin fracture by Bishara [33]. The higher scores indicate fractures that are closer to the adhesive-bracket interface. The ARI scores were broadly similar for the two debonding methods tested; both methods showed high frequencies of scores in the 3 to 5 range, which suggested that debonding with either the debonding pliers or the enamel chisel caused the bond to fail at the bracket-adhesive interface or within the adhesive. This location can be advantageous for the clinician. When the bond is broken at the adhesive-enamel interface, the enamel may fracture during debonding [35].

**Fig. 5:**
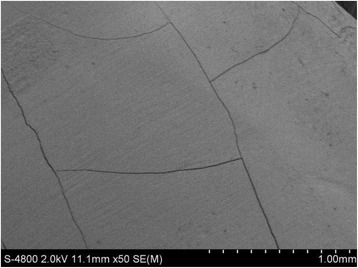
Representative scanning electron microscope (SEM) image of cracks on the enamel surface after debonding with an enamel chisel. SEM magnification: 50×

**Fig. 6:**
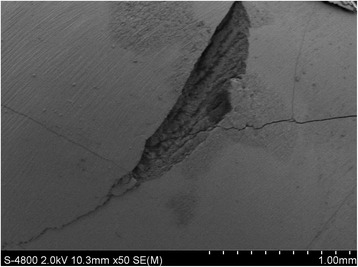
Scanning electron microscope (SEM) image of a fracture on the enamel surface after debonding with an enamel chisel. SEM magnification: 50×

ARI can be used to reflect the fracture position but not to record any damage on the enamel surface. In order to test damage to the enamels, more studies are needed [36, 37]. Based on SEM observations at 50× magnification, 5 out of 9 teeth in the enamel chisel group exhibited cracks on the enamel surface after removing the remnant adhesive (Fig. 5). Moreover, 1 out of 5 cracked teeth also exhibited enamel fracture (Fig. 6). These types of enamel damage were not observed in any of the teeth in the debonding pliers group. Bishara suggested that excessive debonding force (>11.3 MPa) may cause enamel cracks [13]. In this study, for the debonding pliers group, we place the blades of the pliers the area between the wings and the base of the bracket (wings model), but not between the base of the brackets and the enamel surface (base model). According to the investigation by Brosh and colleagues [7], the moment that develops by drawing the point of application away from the bracket-adhesive-enamel interface decreases the force required to detach the bracket. Consequently, lower stress is applied in the wings model, and no cracks on the enamel surface were observed in pliers group. While, the enamel chisel mainly requires impact forces to detach the brackets, and large, instantaneous impact forces may case enamel damage.

### Cleanup process

A roughened enamel surface may facilitate bacterial plaque retention, which produces superficial staining and gingival inflammation. Moreover, the acidic byproduct initiated by a bacterial plaque results in a lower pH, which leads to the chemical dissolution of mineralized hard tissue, and promotes dental caries [38]. Therefore, a smooth tooth surface is important, both for aesthetic reasons, and for resisting demineralization. The purpose of the secondary cleanup process is to restore the natural smooth enamel surface after removing directly bonded brackets in orthodontic treatments.

The diamond-finishing bur and tungsten carbide bur were previously reported to be used for removing adhesive. But burs should not be used alone in orthodontic debonding processes. Abrasion marks by burs increase the roughness on the enamel surface. Therefore, a secondary polishing instrument should be used later [26, 27]. Tungsten carbide bur is a more common instrument for debonding. Based on the review of previous literature, it might produce parallel grooves during the debonding of orthodontic attachments [4, 24, 25]. This clinic characteristic of the diamond bur was similar to tungsten carbide bur [14, 24, 26], and the diamond bur has higher efficiency. In this study, we selected diamond bur as a representative to study the effects of high-speed rotating instrument to enamel surface. Consistent with other studies [4, 25], we found that the least time was spent with the diamond bur + One-Gloss method. However, the enamel appearance was the worst in the diamond bur + One-Gloss method by evaluation of the SEM and surface roughness, compared to the other polishing processes. Indiamond bur + One-Gloss group, the deep grooves and scars caused by diamond-finishing bur could not be reduced or cleared with the One-Gloss polisher. Ye reported that the colour change produced when RMGIC was polished with a carbide bur + One-Gloss was less pronounced than that produced with a carbide bur alone, but significantly more pronounced than that produced with a carbide bur + Sof-Lex or with the PoGo polisher under the same conditions [28]. Those findings suggested that One-Gloss polishers only provided limited improvement over a finishing bur, and that the various combinations of finishing bur and silicone particle polishers were inadequate for removing adhesive. The finding was also consistent with observation from Odaira [22].

In the One-Gloss polisher group, it produced the smoothest surface, with very few shallow scratches. Enamel surfaces after polishing were closest to the original enamel surface by SEM. But it also required the longest operating time, and thus, it exhibited the least efficiency in three cleanup processes tested. Previous studies reported that Sof-Lex, an aluminium-oxide-coated abrasive disk, which is similar to Super-Snap, produced surfaces with the closest appearance to the original enamel surface [4, 39]. In the present investigation, the micro-morphological images of surfaces by Super-Snap exhibited some deep scars, apart from the shallow scratches, but the result was far superior to that produced with the diamond bur + One-Gloss. The process by Super-Snap only required moderate operating times. The operating times observed in our study were much longer than those reported in other previous investigations [4, 25]. This difference might result from the fact that we only applied light pressure when polishing.

We used SEM images to gain a better understanding of how the enamel surfaces changed with different adhesive removal methods. However, SEM requires subjective inspection, and cannot be used for comparative assessments alone [40]. Alternatively, quantitative techniques, such as surface roughness tester, can provide more reliable comparisons of different cleanup processes. The Ra value reflects the overall roughness of the enamel surface, as shown in previous studies [4, 19, 39]. However, the Ra value does not fully describe surface roughness [41, 42], because it cannot indicate the depth of irregularities or differentiate between shallow and deep grooves [4]. Therefore, we introduced an additional variable, Rz, to evaluate the degree of scratching caused by polishing. Rz was defined as the average distance from the mean line to the five highest peaks and the five deepest valleys. The Rz value is more stable than the distance between the highest and lowest points (Rt or Rmax).

According to the statistical results of ARI, both debonding methods showed high frequencies of ARI scores in the 3 to 5 range and the chi-square test showed no difference between two groups. These results suggested that the amount of adhesive remaining on tooth surface by two debonding methods was similar before cleanup process. So we combined the samples in different debonding groups with same cleanup procedures for statistical analysis of surface roughness measurement. The results showed that the diamond bur + One-Gloss produced the roughest surfaces. The Ra and Rz values were 0.443 ± 0.172 μm and 2.202 ± 0.791 μm, respectively. These values were significantly higher than those in the other two polishing methods. The SEM observations showed similar results. Previous studies reported Ra values of 0.125–0.260 μm for nanofilled composites polished with Al_2_O_3_ abrasive grain discs [43, 44]. In the present study, the Ra value after polishing with Super-Snap was 0.141 ± 0.073 μm, which was close to the value achieved with the Al_2_O_3_ discs, but lower than that reported in other studies that tested on enamel surfaces [4]. The One-Gloss polisher exhibited the least roughness, which showed no significant difference from the intact enamel surface, both in Ra and Rz values. The Super-Snap method also produced an Ra value similar to that of the intact enamel surface, but the Rz value was significantly higher than those observed with the One-Gloss method and the control. This implied that cleanup with Super-Snap produced an acceptable enamel surface, but it left a number of deep scratches. These findings were confirmed in the SEM images. The results of our study were based on the removal of RMGIC. Due to the different bonding properties of RMGIC and resin adhesive [30, 31], our data may not accurately reflect the actual situation of the removal of the resin adhesive from enamel. The comparison study of debonding different materials such as RMGIC and resin might be done in the future.

## Conclusions

On the basis of our investigation, the following conclusions could be drawn:

1. Debonding with debonding pliers can reduce the risk of enamel cracks; therefore, this method is safer than the enamel chisel method for removing brackets.
2. Diamond finishing burs produced the worst enamel appearances, and the deep grooves and scars caused by the finishing bur could not be reduced or smoothed with a One-Gloss polisher.
3. Cleanup with Super-Snap produced acceptable results, but a number of deep scratches were left on the enamel surfaces.
4. One-Gloss silicone particle polishing alone produced enamel surfaces that were closest to the intact enamel surface, but the polishing efficiency was the lowest of all the methods tested.

## Abbreviations

ANOVA: Analysis of variance
ARI: Adhesive remnant index
Ra: Average roughness
RMGIC: Resin modified glass ionomer cement
Rz: 10-point height of irregularities
SEM: Scanning electron microscope

## References

[1] Buonocore MG (1955). A simple method of increasing the adhesion of acrylic filling materials to enamel surfaces. J Dent Res.

[2] Newman GV (1965). Epoxy adhesives for orthodontic attachments: progress report. Am J Orthod.

[3] Shuler FS, van Waes H (2003). SEM-evaluation of enamel surface after removal of fixed orthodontic appliances. Am J Dent.

[4] Ozer T, Basaran G, Kama JD (2010). Surface roughness of the restored enamel after orthodontic treatment. Am J Orthod Dentofac Orthop.

[5] Dovgan JS, Walton RE, Bishara SE (1995). Electrothermal debracketing: patient acceptance and effects on the dental pulp. Am J Orthod Dentofac Orthop.

[6] Ireland AJ, Hosein I, Sherriff M (2005). Enamel loss at bond-up, debond and clean-up following the use of a conventional light-cured composite and a resin-modified glass polyalkenoate cement. Eur J Orthod.

[7] Brosh T, Kaufman A, Balabanovsky A, Vardimon AD (2005). In vivo debonding strength and enamel damage in two orthodontic debonding methods. J Biomech.

[8] Su MZ, Lai EH, Chang JZ, Chen HJ, Chang FH, Chiang YC (2012). Effect of simulated debracketing on enamel damage. J Formos Med Assoc.

[9] Larmour CJ, MeCabe JF, Gordon PH (1998). An ex vitro investigation into the effects of chemical solvence on the debond behaviour of ceramic orthodontic brackets. Br J Orthod.

[10] Boyer DB, Engelhardt G, Bishara SE (1995). Debonding orthodontic ceramic brackets by ultrasonic instrumentation. Am J Orthod Dentofac Orthop.

[11] Ma T, Marangoni RD, Flint W (1997). In vitro comparison of debonding force and intrapulpal temperature changes during ceramic orthodontic bracket removal using a carbon dioxide laser. Am J Orthod Dentofac Orthop.

[12] Olszowska JJ, Tandecka K, Szatkiewicz T, Sporniak-Tutak K, Grocholewicz K (2014). Three-dimensional quantitative analysis of adhesive remnants and enamel loss resulting from debonding orthodontic molar tubes. Head Face Med.

[13] Bishara SE, Fonseca JM, Boyer DB (1995). The use of debonding pliers in the removal of ceramic brackets: force levels and enamel cracks. Am J Orthod Dentofac Orthop.

[14] Zarrinnia K, Eid NM, Kehoe MJ (1995). The effect of different debonding techniques on the enamel surface: An in vitro qualitative study. Am J Orthod Dentofac Orthop.

[15] Oliver RG, Griffiths J (1992). Different techniques of residual composite removal following debonding time taken and surface enamel appearance. Br J Orthod.

[16] Hosein I, Sherriff M, Ireland AJ (2004). Enamel loss during bonding, debonding, and cleanup with use of a self-etching primer. Am J Orthod Dentofac Orthop.

[17] Burapavong V, Marshall GW, Apfel DA, Perry HT (1978). Enamel surface characteristics on removal of bonded orthodontic brackets. Am J Orthod.

[18] Olszowska JJ, Tandecka K, Szatkiewicz T, Stepien P, Sporniak-Tutak K, Grocholewicz K (2015). Three-dimensional analysis of enamel surface alteration resulting from orthodontic clean-up comparison of three different tools. BMC Oral Health.

[19] Kim SS, Park WK, Son WS, Ahn HS, Ro JH, Kim YD (2007). Enamel surface evaluation after removal of orthodontic composite remnants by intraoral sandblasting: A 3-dimensional surface profilometry study. Am J Orthod Dentofac Orthop.

[20] Bonetti GA, Zanarini M, Parenti S, Lattuca M, Marchionni S, Gatto MR (2011). Evaluation of enamel surfaces after bracket debonding: An in-vivo study with scanning electron microscopy. Am J Orthod Dentofac Orthop.

[21] Chen L, Zhang JJ, Zhao BJ (2011). Research of three adhesive removal methods influences on tooth surface after metal brackets debonding. Chinese J Aesthet Med.

[22] Odaira C, Itoh S, Ishibashi K (2011). Clinical evaluation of a dental color analysis system: the crystaleye Spectrophotometer. J Prosthodont Res.

[23] Alexander R, Xie J, Fried D (2002). Selective removal of residual composite from dental enamel surfaces using the third harmonic of a Q-switched ND:YAG laser. Lasers Surg Med.

[24] Campbell PM (1995). Enamel surfaces after orthodontic bracket debonding. Angle Orthod.

[25] Eminkahyagil N, Arman A, Cetinsahin A, Karabulut E (2006). Effect of resin-removal methods on enamel and shear bond strength of rebonded brackets. Angle Orthod.

[26] Bishara SE, Trulove TS (1990). Comparisons of different debonding techniques for ceramic brackets: an in vitro study. Part II. Findings and clinical implications. Am J Orthod Dentofac Orthop.

[27] Eliades T, Kakaboura A, Eliades G, Brantley TG (2001). Comparison of enamel colour changes associated with orthodontic bonding using two different adhesive. Eur J Orthod.

[28] Ye C, Zhao Z, Zhao Q, Du X, Ye J, Wei X (2013). Comparison of enamel discoloration associated with bonding with three different orthodontic adhesives and cleaning-up with four different procedures. J Dent.

[29] Tufekci E, Merrill TE, Pintado MR, Beyer JP, Brantley WA (2004). Enamel loss associated with orthodontic adhesive removal on teeth with white spot lesions: An in vitro study. Am J Orthod Dentofac Orthop.

[30] Silverman E, Cohen MS, Demke RS, Silverman M (1995). A new light-cured glass ionomer cement that bonds brackets to teeth without etching in the presence of saliva. Am J Orthod Dentofac Orthop.

[31] Sfondrini MF, Cacciafesta V, Pistorio A, Sfondrini G (2001). Effects of conventional and high-intensity light-curing on enamel shear bond strength of composite resin and resin-modified glass-ionomer. Am J Orthod Dentofac Orthop.

[32] International Organization for Standardization. Dental Materials Guidance on Testing on Adhesion to Tooth Structure. Geneva, Switzerland ISO TR 11405. 2004.

[33] Bishara SE, Gordan VV, VonWald L, Jakobsen JR (1999). Shear bond strength of composite, glass ionomer, and acidic primer adhesive systems. Am J Orthod Dentofac Orthop.

[34] Stout KJ (1981). Surface roughness: measurement, interpretation and significance of data. Mater Eng.

[35] Bishara SE, Gordan VV, VonWald L, Olson ME (1998). Effect of an acidic primer on the shear bond strength of orthodontic brackets. Am J Orthod Dentofac Orthop.

[36] Eslamian L, Borzabadi-Farahani A, Mousavi N, Ghasemi A (2012). A comparative study of shear bond strength between metal and ceramic brackets and artificially aged composite restorations using different surface treatments. Eur J Orthod.

[37] Eslamian L, Borzabadi-Farahani A, Tavakol P, Tavakol A, Amini N, Lynch E (2015). Effect of multiple debonding sequences on shear bond strength of new stainless steel brackets. J Orthod Sci.

[38] Reis AF, Giannini M, Lovadino JR, dos Santos Dias CT (2002). The effect of six polishing systems on the surface roughness of two packable resin-based composites. Am J Dent.

[39] Janus J, Fauxpoint G, Arntz Y, Pelletier H, Etienne O (2010). Surface roughness and morphology of three nanocomposites after two different polishing treatments by a multitechnique approach. Dent Mater.

[40] Eliades T, Gioka C, Eliades G, Makou M (2004). Enamel surface roughness following debonding using two resin grinding methods. Eur J Orthod.

[41] Grossman ES, Rosen M, Cleaton-Jones PE, Volchansky A (2004). Scientific surface roughness values for resin based materials. SADJ.

[42] Marigo L, Rizzi M, La Torre G, Rumi G (2001). 3-D surface profile analysis: different finishing methods for resin composites. Oper Dent.

[43] Senawongse P, Pongprueksa P (2007). Surface roughness of nanofill and nanohybrid resin composites after polishing and brushing. J Esthet Restor Dent.

[44] Attar N (2007). The effect of finishing and polishing procedures on the surface roughness of composite resin materials. J Contemp Dent Pract.

